# Comparison of Small RNA Profiles of *Glycine max* and *Glycine soja* at Early Developmental Stages

**DOI:** 10.3390/ijms17122043

**Published:** 2016-12-06

**Authors:** Yuzhe Sun, Zeta Mui, Xuan Liu, Aldrin Kay-Yuen Yim, Hao Qin, Fuk-Ling Wong, Ting-Fung Chan, Siu-Ming Yiu, Hon-Ming Lam, Boon Leong Lim

**Affiliations:** 1 School of Biological Sciences, The University of Hong Kong, Pokfulam, Hong Kong, China; yzsun@hku.hk; 2 School of Life Sciences, The Chinese University of Hong Kong, Shatin, Hong Kong, China; zeta.mui@gmail.com (Z.M.); aldrinlaurent@gmail.com (A.K.-Y.Y.); qinhao13@gmail.com (H.Q.); wongfl@gmail.com (F.-L.W.); tf.chan@cuhk.edu.hk (T.-F.C.); 3 Center for Soybean Research of the Partner State Key Laboratory of Agrobiotechnology, The Chinese University of Hong Kong, Shatin, Hong Kong, China; 4 Department of Computer Science, The University of Hong Kong, Pokfulam, Hong Kong, China; liuxuan@connect.hku.hk (X.L.); smyiu@cs.hku.hk (S.-M.Y.)

**Keywords:** miRNA, phasiRNA, *Glycine max*, *Glycine soja*, sliding window analysis, *PHAS* loci, *PHAS*-phasiRNA pathway

## Abstract

Small RNAs, including microRNAs (miRNAs) and phased small interfering RNAs (phasiRNAs; from *PHAS* loci), play key roles in plant development. Cultivated soybean, *Glycine max*, contributes a great deal to food production, but, compared to its wild kin, *Glycine soja*, it may lose some genetic information during domestication. In this work, we analyzed the sRNA profiles of different tissues in both cultivated (C08) and wild soybeans (W05) at three stages of development. A total of 443 known miRNAs and 15 novel miRNAs showed varying abundances between different samples, but the miRNA profiles were generally similar in both accessions. Based on a sliding window analysis workflow that we developed, 50 *PHAS* loci generating 55 21-nucleotide phasiRNAs were identified in C08, and 46 phasiRNAs from 41 *PHAS* loci were identified in W05. In germinated seedlings, phasiRNAs were more abundant in C08 than in W05. Disease resistant *TIR-NB-LRR* genes constitute a very large family of *PHAS* loci. PhasiRNAs were also generated from several loci that encode for NAC transcription factors, Dicer-like 2 (DCL2), Pentatricopeptide Repeat (PPR), and Auxin Signaling F-box 3 (AFB3) proteins. To investigate the possible involvement of miRNAs in initiating the *PHAS*-phasiRNA pathway, miRNA target predictions were performed and 17 C08 miRNAs and 15 W05 miRNAs were predicted to trigger phasiRNAs biogenesis. In summary, we provide a comprehensive description of the sRNA profiles of wild versus cultivated soybeans, and discuss the possible roles of sRNAs during soybean germination.

## 1. Introduction

Small RNAs (sRNA) are important regulators of plant development and physiology. There are two main categories of sRNAs: Noncoding microRNAs (miRNA) and small interfering RNAs (siRNA) [1]. In plants, mature miRNAs are 20- to 22-nucleotide (nt) single-stranded RNAs that regulate target mRNA expression by cleavage or translational inhibition [2,3,4]. While most of the cleaved transcripts are degraded, some generate 21-nt secondary phased siRNAs [2,5]. In previous studies regarding these siRNAs, phased siRNAs that were not experimentally validated to be *trans*-acting were termed “phasiRNAs“, while those found to be *trans*-acting were named “*trans*-acting siRNAs” (tasiRNAs) [6]. The loci that generate these phasiRNAs are called *PHAS* loci. These loci are also protein-coding, producing secondary *cis*-acting siRNAs that regulate themselves. In contrast, the loci that generate tasiRNAs are non-coding, and are termed *Trans-Acting SiRNA* (*TAS*) loci [6]. These *TAS* loci produce secondary siRNAs that target protein-coding genes found elsewhere in the genome. In *Arabidopsis thaliana*, some tasiRNAs are generated by the cleavage of the noncoding *TAS* transcripts (*TAS1/2/3/4*) [2,5,7], which act in *trans* in cleaving target mRNAs. Currently, at least four *TAS* loci have been identified in *Arabidopsis*. Argonaute 1 (AGO1)-miR173 initiates tasiRNA formation in *TAS1* and *TAS2* transcripts, and the generated tasiRNAs primarily target *PPR* protein family transcripts, which are involved in RNA editing in chloroplasts and mitochondria [8,9,10]. *TAS3* is triggered by miR390 and its phasiRNA has been experimentally proven to slow down a plant’s transition from juvenile to adult by targeting auxin responsive factors (*ARFs*) [11]. *TAS4* is triggered by miR828 and then one of its tasiRNAs targets, *MYB113*, regulates the anthocyanin production pathway [12]. Additionally, miR482-triggered *TAS5* was found in tomatoes and dual miR156 and/or miR529-mediated *TAS6* was found in *Physcomitrella patens*. Nonetheless, the production of phasiRNA/tasiRNA requires the involvement of miRNA: Some AGO1/7-bound miRNAs triggers the cleavage of *PHAS* or *TAS* mRNAs into 5’ and 3’ transcript fragments. Subsequently, multiple proteins participate in this process, including AGO1/7, suppressor of gene silencing 3 (SGS3), RNA-dependent RNA polymerase 6 (RDR6), and DCL proteins to form a series of phasiRNAs [13,14,15,16]. Either the 5’ or the 3’ fragments are then converted into double-stranded RNAs (dsRNAs) by RDR6 to form dsRNA precursors. Finally, DCL proteins will cleave the dsRNA precursor in a phased pattern, starting from the miRNA-guided cleavage site [6]. The cleaved fragments are oriented head to tail and all have precisely the same length. phasiRNAs repress the target mRNA by translational inhibition or by causing the degradation of target mRNAs [17,18,19,20].

Cultivated soybean (*Glycine max*) is an important crop for the production of edible oil and as a source of dietary protein [21,22]. As soybeans are strict cleistogamic pollinators, genomic variation may largely be reduced. The genetic homogeneity of this plant has been further intensified by cultivation. Wild soybeans (*Glycine soja*), on the other hand, may have retained some genetic traits that disappeared from the cultivated varieties after domestication. However, previous studies showed that the *G. soja* genome contains consensus sequences that represented a 97.65% coverage of *G. max* genome sequences [23]. However, the two soybean subspecies exhibit strong morphological diversity (seed color, seed size, leaf shape, etc.) and exhibit different responses to biotic and abiotic stress [21,24]. To date, hundreds of miRNAs and siRNAs have been identified on a genome-wide scale in soybeans [21,25,26]. In order to compare sRNA profiles between cultivated and wild soybeans, and to find potential sRNA transcriptional determinants that contributed to the phenotypic differences, we analyzed 14 small RNA libraries, created from different tissues across developmental stages from both cultivated soybeans (C08) and wild soybeans (W05). A strategy to identify *PHAS* loci and phasiRNA using a slide-window workflow was also developed. Using this method, differential phasiRNA profiles were observed in various developmental stages of both cultivated and wild soybeans.

## 2. Results

### 2.1. Construction of Small RNA Libraries from Glycine max and Glycine soja

Fourteen sRNA libraries were constructed from cultivated soybean accession C08 (*Glycine max*) and its wild progenitor W05 (*Glycine soja*), including roots and aerial parts. Root samples were collected from germinated seedlings (gr), young seedlings (yr), and seedlings (sr). Aerial part tissues, which included the cotyledons, were collected from germinated seedlings (ga), young seedlings (ya), and seedlings (sp). Additionally, trifoliate samples were collected from soybean seedlings (st) (Table S1).

Small RNA reads in 18 to 30 nucleotides (nt) range were retained. After removal of low quality and adaptor sequences, a total of 85,327,846 and 86,812,643 clean reads were yielded from C08 and W05 libraries, respectively (Table S1). The reads were then mapped to either single or multiple positions in the genome. In total, 67,855,161 and 63,974,900 mapped reads (79.5% and 73.7% of the total) were retained in C08 and W05, respectively, corresponding to 11,175,309 and 9,894,780 unique sequences (Table S1). The length distribution of mapped reads revealed that the population of small RNAs in different size clusters varied among different tissues (Figure S1). sRNAs 20–24 nt in size were the most abundant, which was consistent with a former report [27]. By comparing the 20–24 nt sRNA profiles, it was shown that W05 was particularly rich in 24 nt sRNA (Figure S1).

### 2.2. Identification of Novel miRNAs and Differential miRNA Expression in Glycine max and Glycine soja

As only 2.35% of the total genome sequences are different between *G. max* and *G. soja* [23], the *G. max* genome (Wm82.a2.v1) was used as reference in miRNA identification and *PHAS* loci and phasiRNA prediction. Because gene location and annotation are different in these two genomes, it is difficult to integrate miRNA information with phasiRNA comparison. Although the mapping of *G. soja* libraries to the *G. max* genome leads to the loss of some sRNA information specific to *G. soja*, it helps to identify differential expression of sRNA between the two species.

A total of 621 known miRNAs (Table S2a) and 15 novel miRNAs (Table S3) were found. We also analyzed miRNA differential expression. In total, 443 known miRNAs and 15 novel miRNAs were found to be differentially accumulated in at least two pairwise comparisons (Tables S2b and S3).

**Figure 1:**
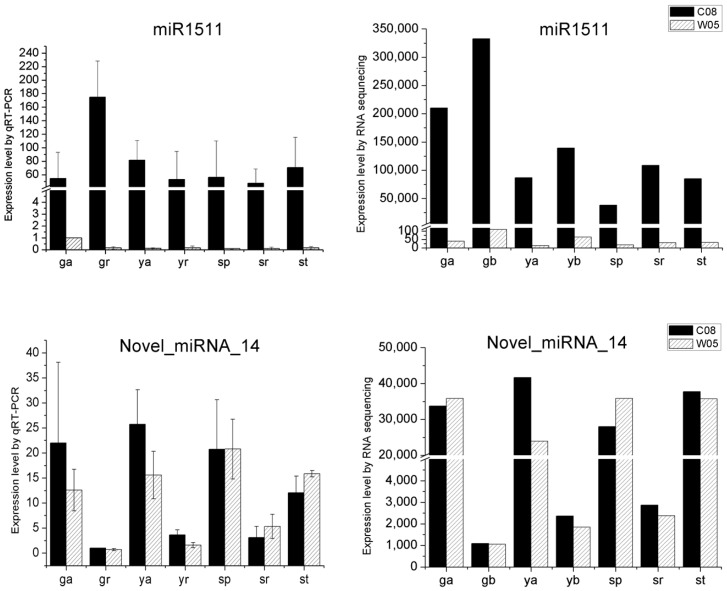

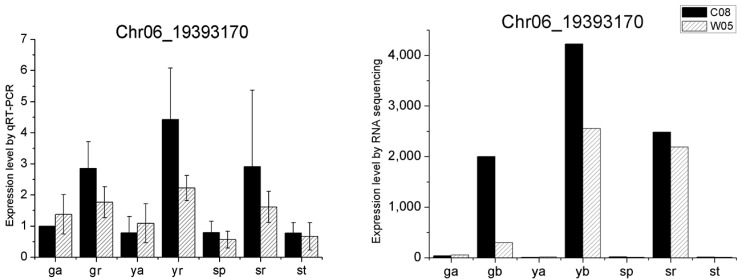
Validation of differential expressions of sRNAs. The differential expressions of miRNAs and phasiRNAs identified by sRNA sequencing (**right**) were validated by qRT-PCR (**left**). *Y*-axis showed the normalized reads of sRNA in 14 samples (**right**) and relative expression levels (**left**). Error bar shows the standard deviation (SD) of the three biological replicates used in qRT-PCR.

Using psRNATarget, most of the novel miRNAs were predicted to either target transcription factors or development related proteins (Table S3). Embryo sac development arrest 14 (EDA 14) and F-box family proteins are strongly related to soybean development, especially seed germination. On the other hand, GRAS family transcription factor, targets of which were highly accumulated in young seedling roots and seedling roots, regulates plant development in root patterning [28]. Novel_miRNA_1, Novel_miRNA_9, and Novel_miRNA_15 targeted NAC transcription factors, which are responsive to biotic and abiotic stress responses [29]. Relative expressions of novel_miRNA_14 in 14 samples were further validated by qRT-PCR (Figure 1). The results were consistent between qRT-PCR and sRNA sequencing.

### 2.3. Tissue Types and Developmental Stages Are Significant Determinants of miRNA Profiles for Both Wild and Cultivated Soybeans

**Figure 2:**
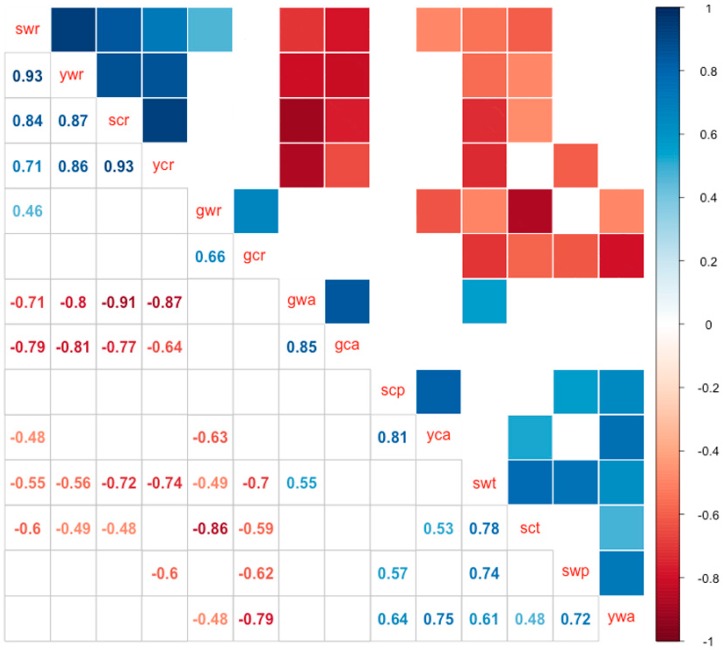
Pairwise correlation matrix of differentially-expressed miRNAs across tissue types, developmental stages, and wild and cultivated accessions. Pearson correlations between pairwise samples among all 14 experimental conditions were calculated by considering differentially-expressed miRNAs between each pairwise comparison as the distance between the two conditions. Blue boxes indicate positive correlation values (0 ≤ *r* ≤ 1; with 1 being complete positive correlation) and red boxes represent negative correlation values (−1 ≤ *r* ≤ 0, with −1 being complete anti-correlation) between samples. Correlation with *p*-values >0.1 are not shown in this matrix. The order of the rows was determined by the hierarchical clustering of the correlation matrix. Closely-related conditions, such as conditions with the same type of tissues, were therefore grouped together. “gca” and “gcr”: aerial part and roots, respectively, of germinated seedlings of C08; “yca” and “ycr”: aerial part and roots, respectively, of young seedlings of C08; “sct”: trifoliates of C08 seedlings; “scp” and “scr”: Primary leaves and roots, respectively, of C08; “gwa” and “gwr”: aerial part and roots, respectively, of germinated seedlings of W05; “ywa” and “ywr”: Aerial part and roots, respectively, of young seedlings of W05; “swt”: Trifoliates of W05 seedlings; “swp” and “swr”: Primary leaves and roots, respectively, of W05.

The correlation matrix showed that there are strong correlations among the roots of young seedlings and seedlings in both wild and cultivated soybeans, as indicated by the cluster of blue squares in the upper left corner (gwr vs. gcr) (Figure 2). A strong correlation was also found between the roots and the aerial parts of germinated seedlings of wild and cultivated soybeans (gwa vs. gca). In general, all seven C08 samples were positively correlated with the corresponding W05 samples of the same developmental stage or tissue type, which indicated that there were no fundamental differences in miRNA expression between C08 and W05. Two samples of W05, the trifoliate and primary leaves, showed substantial correlations (Figure 2).

To further investigate if there were significant differences between C08 and W05, we calculated the fold-change (log_2_) in miRNA abundances in samples from W05 against corresponding samples from C08 (Figure S2). miRNAs with less than a two-fold difference (W05/C08) in abundance were removed. miRNAs in germinated seedlings were more diverse than those in other developmental stages, which implied that miRNA profile differences between C08 and W05 were largely in germinated seedlings. Particularly, miR1511 was dramatically enriched in all the tissues of C08, and, for miR156r, its abundance was higher in C08, in all samples except seedling trifoliates (swt_sct) (Figure S2).

### 2.4. miRNA Profiles

**Figure 3:**
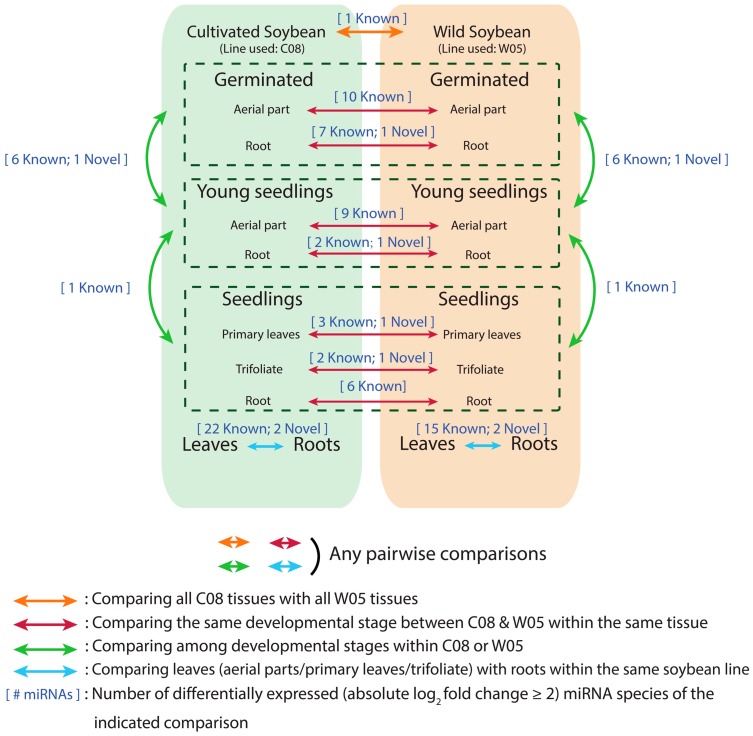
Differential miRNA expressions between different samples. We studied miRNAs with >3000 counts in seven samples of C08 or W05. Differential expression analysis was performed between C08 samples and W05 samples (orange and red arrows), between leaf samples and root samples (blue arrows), and between developmental stages (green arrows). Details of these miRNAs are presented in Table S4.

miRNAs with total reads >3000 reads in seven samples of C08 or W05 were studied (Figure 3, Table S4). Ten known miRNAs were differentially accumulated between C08 and W05 cotyledons (Figure 3, Table S4a) in which only miR1510a/b, miR1511, miR156r, and miR5670b had high counts. While miR1511 had 210,228 counts in C08 cotyledons, only 40 counts were found in W05, which indicated that mRNA regulation involving miR1511 had been initiated in C08 but not in W05. miR156r and miR5670 also had a much higher abundance in C08 cotyledons than in W05 cotyledons. By contrast, miR1510a/b abundance was more than seven-fold higher in W05 than in C08 (Table S4a). However, when we examined seven known miRNAs and Novel_miRNA_2 that were differentially accumulated between C08 and W05 germinating roots, the normalized absolute counts of miR156r and miR5670 were low (Table S4b). Nevertheless, miR1511 maintained high abundance in C08 roots like in C08 cotyledons, and the fold change (W05/C08) of miR1510b-5p was high (Table S4b). Twenty-two known miRNAs and two novel miRNAs in C08 were differentially accumulated between cotyledons and roots; 15 known miRNAs and two novel miRNAs in W05 were differentially accumulated between cotyledons and roots (Figure 3, Table S4c). Seventeen miRNAs in C08 were highly accumulated in leaves and seven in roots, while 10 miRNAs in W05 were highly accumulated in leaves and seven in roots (Table S4c). Two novel miRNAs, Novel_miRNA_11 and Novel_miRNA_14, were found in both C08 and W05 (Table S4c).

Changes in miRNA levels were also compared between developmental stages under our growth conditions. Six known miRNAs and Novel_miRNA_2 were commonly and differentially accumulated between the four germinated seedling samples and four young seedling samples (Figure 3, Table S4d). Additionally, only miR2109-5p had common and differential abundances between young seedling samples and seedling samples (Figure 3, Table S4e).

### 2.5. Genome-Wide Identification of PHAS Loci and PhasiRNAs

**Figure 4:**
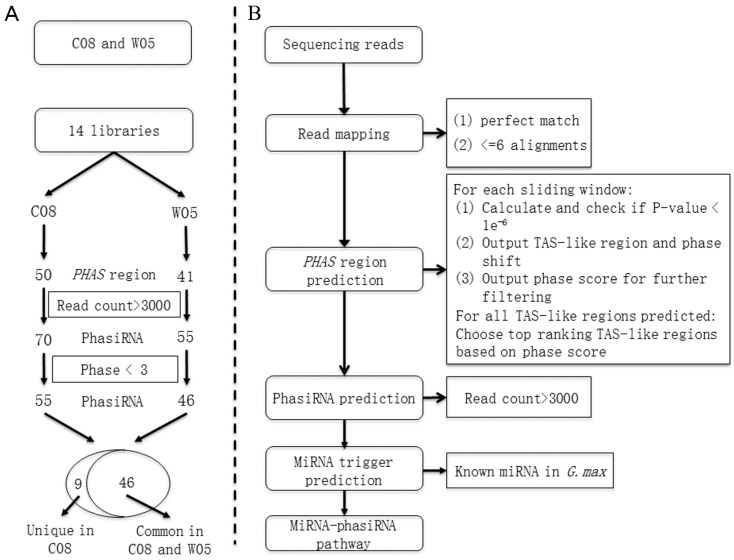
The pipeline for phasiRNA predictions: (**A**) The workflow for the comparisons between the phasiRNA profiles of C08 and W05. The numbers of common and unique phasiRNAs are shown in the Venn diagram; (**B**) The workflow for phasiRNA identification. Sequencing reads were mapped to the reference genome Gmax_275_Wm82.a2.v1 and only those reads that could be mapped to at most six positions on the reference genome with perfect matches were used. A sliding-window method was then applied to predict genome-wide PHAS regions using the pattern of mapped reads based on the ranking of *p*-values and phase scores. In each predicted *PHAS* region, loci with on-phase high-count reads (generating phasiRNAs >3000 total reads in seven sample of each variety) were selected and regarded as putative phasiRNA-generating loci. A characterization of each putative phasiRNA was performed to identify any possible miRNA triggers that initiated the biogenesis of phasiRNAs. The final product was a proposed pathway for each miRNA–phasiRNA pair.

Twenty-one-nucleotide phasiRNAs, triggered by miRNAs, are a typical feature of phasiRNA initiation [30]. In this study, a pipeline has been developed for genome-wide identification of the *PHAS* loci, phasiRNAs, and miRNA–phasiRNA pathways. The pipeline started with high-throughput next-generation RNA sequencing data that then went through multiple filtering stages (Figure 4). Using this sliding window method, 50 and 41 *PHAS* loci were predicted in C08 and W05, respectively, at a stringent threshold *p* value of <1 × 10^−6^ (Table 1, Figure 4). After mapping, all the phasiRNAs with a total count >3000 in the seven samples of each variety were retained. Seventy-one and 55 phasiRNAs were identified in C08 and W05 libraries, respectively. Phase graphs were then plotted for each locus, and those loci with more than two phases of phasiRNAs were discarded (Figures S3 and S4). Finally, 55 phasiRNAs 21-nt in length from C08 and 46 phasiRNAs 21-nt in length from W05 libraries, met the above criteria (Table S5).

Considering that phasiRNAs are triggered by miRNA, target prediction of miRNAs was performed. A total of 5716 binding sites were predicted. Binding sites that were within 420 nt (20 × 21 nt) of the phasiRNAs were retained. Fifty-two phasiRNAs in C08 were predicted to be triggered by 17 miRNAs and 50 phasiRNAs in W05 were predicted to be triggered by 15 miRNAs (Table S6a). Fourteen miRNAs were predicted to dually trigger the biogenesis of phasiRNAs in C08 and W05. miR2118, miR390, and miR9761 were only found in C08, and miR1536 could only trigger phasiRNAs in W05 (Table S6a). Furthermore, four miRNAs (miR1507, miR1509, miR1510, and miR1514) in C08 and five miRNAs (miR1508, miR1510, miR1514, miR393, and miR5674) in W05 were predicted to trigger more than one *PHAS* locus. Interestingly, miR1510 can target multiple *PHAS* loci, including three regions in C08 and four regions in W05 (Table S6b and Table S6c). The abundances of three sRNAs, miR1511, novel_miRNA_14, and Chr06_19393170, were validated by qRT-PCR in 14 soybean samples (Figure 1). Their expression patterns corresponded closely with the RNA sequencing data.

### 2.6. Differential Expression of PhasiRNA in Different Tissues

A number of phasiRNAs were differentially or preferentially accumulated in some tissues (Table 2). In germinated seedling roots, most of the phasiRNAs had lower reads in W05 than in C08, except the phasiRNAs from *Glyma.13G187900*, *Glyma.07G048000*, and *Glyma.16G147100*. Similarly, in seedling trifoliates, most of the phasiRNAs had lower reads in W05 than in C08 except phasiRNAs from the two *PHAS* genes (Table 2). In all seven tissues, eight phasiRNAs from *Glyma.13G187900* and *Glyma.07G048000*, had higher abundance in W05. Furthermore, one phasiRNA from *AFB3* had higher reads in the cotyledons of W05 (Table 2).

Dramatic differences in phasiRNA abundances could be seen between the aerial parts and roots across all three development stages of both accessions. The phasiRNAs shown in green were preferentially accumulated in leaves and the phasiRNAs shown in red were preferentially accumulated in roots (Table 3). The preferences were independent of developmental stages and accessions. Interestingly, three *TIR-NB-LRR* loci (*Glyma.04G219600*, *Glyma.06G285500*, and *Glyma.12G163000*) that produce phasiRNAs were preferentially accumulated in leaves, whereas another *TIR-NB-LRR* locus (*Glyma.06G205100*) that produces phasiRNAs was accumulated almost exclusively in roots (Table 3).

In addition, four phasiRNAs from *Chr15_44546856_44547257* and five phasiRNAs from *Glyma.09G032400* showed higher abundances in aerial parts at all three stages (Table 3). A phasiRNA generated from a *PPR* locus is highly accumulated in leaves at all three stages (Table 3). PPR proteins participate in post-transcriptional processes including RNA editing, which occurs in plastids and mitochondria [10,31]. Leaf cells have a large number of chloroplasts while roots cells only have plastids. Hence, the higher abundance of this phasiRNA in leaves may be required to control the higher PPR transcript abundances in the leaves. However, one phasiRNA from an *AFB3* locus (*Glyma.16G050500*) had elevated abundance in roots (Table 3).

Additionally, nine phasiRNAs were exclusively found in C08 (Table S5d). These nine phasiRNAs belonged to a *TIR-NB-LRR* gene, a *NTL9* (NAC domain-containing protein 9-like) gene, a LRR and NB-ARC domain, and five other un-annotated genes (Table S5d). Abundances of two phasiRNAs from the *NTL9* gene gradually increased over the three developmental stages in both leaves and roots. *Chr04_47363101* preferentially enriched in aerial tissues while *Chr06_19393086* from a *TIR-NB-LRR* gene accumulated in roots (Table S5d).

## 3. Discussion

### 3.1. Differential Expressions of sRNAs between Cultivated and Wild Soybean and Their Biological Significance

The sRNA network is an important mechanism that regulates gene expression at transcriptional and post-transcriptional levels. In this study, we focused on the analyses of miRNAs and phasiRNAs in two soybean accessions including seven tissues from three developmental stages. During seed germination, cotyledon is a major tissue in the aerial part of the plant and is the first photosynthetic structure to develop from the hypocotyl. Although a large number of miRNAs were found to be differentially expressed between two individual samples, much fewer miRNAs were universally differentially expressed between accessions (C08 vs. W05), tissues (root vs. leaf) and developmental stages (germinated seedling vs. young seedling vs. seedling). Only miR1511 exhibited significantly different abundances across all seven tissues between the two accessions, implying that the miRNA profiles are similar between C08 and W05 (Table S4f). The abundance of miR1511 was greatly elevated in C08 (3276 times higher than that in W05) during germination (Table S4f). miR1511 has been studied in *G. max* (C08), and *G. soja* (W05) [32,33], in the nodules of *Medicago* [34], and in *Phaseolus vulgaris* [35]. In soybeans, glutamine amidotransferase class-I, which is an important enzyme involved in glutamine biosynthesis, was identified as the target of miR1511 [33]. Here, the abundance of miR1511 was, by orders of magnitude, higher in all the tissue types of C08 than in W05, and its level was twice as high in the roots as in the leaves. Thus, we speculated that miR1511 might be an important factor in modulating nitrogen metabolism in the roots of C08 (Table S4f).

During plant development, the miRNA expression profile changes according to regulatory demand. Several pathways were strongly involved in plant juvenile-to-adult and adult-to-flowering phase transitions. One of the pathways, the age pathway, is mainly dictated by miRNAs. Two key miRNAs, miR156, and miR172, modulate juvenility and flowering. Previous studies showed that miR156 is necessary for maintaining juvenility in plants. The accumulation level of miR156 went down when the plant transitioned into adulthood and later to flowering. At the same time, abundance of miR172 gradually increases when plants enter the flowering (reproductive) phase, acting downstream of the miR156 targets, SPLs (Squamosa Promoter-Binding Protein-Likes). Higher miR172 abundance promotes juvenile-to-adult phase changes and flowering [36,37,38,39,40]. In our data set, miR156 was highly expressed in the cotyledons as well as roots of both C08 and W05, then the abundance declined in the leaves of young seedlings by almost 10 times (Table S4g). However, in C08 seedling leaves, miR156 kept on declining while, in W05 seedling leaves, the abundance retained. Meanwhile, miR172 expression increased dramatically in the young seedling leaves of both C08 and W05 compared to germinated seedling cotyledons, which was consistent with previous reports [41]. However, in the roots of W05, miR172 expression was stable in germinated seedlings and young seedlings, but declined in seedlings. This same decline was also observed in the roots of C08, starting in the young seedling stage, one stage earlier than in W05 (Table S4g). The decline of miR156 abundance and the elevation of miR172 abundance in the leaves of young seedlings could be a signal that suggests the initiation of phase transition. If so, the decline of miR172 abundance in roots happened earlier in C08 than in W05, suggesting that C08 may reach the reproductive growth sooner than W05.

### 3.2. PHAS Loci and phasiRNAs in Soybean

Secondary phasiRNAs are generated from *PHAS* loci. The annotation of the *PHAS* loci in this study showed that 23 out of 50 (46%) in C08 and 17 out of 41 (41.5%) in W05 were *TIR-NB-LRR* disease resistance coding genes (Table 1). The *NB-LRR* class of plant disease resistance (R) proteins has been shown to play an important role in defense signaling [42]. A large number of phasiRNAs were generated from *NB-LRR* loci in *Medicago* and *Solanaceae*, and the production of these phasiRNAs is probably related to plant defense mechanisms [5,43]. Additionally, our miRNA data showed that miR168, of which the target is *AGO1*, was differentially expressed between tissues and between accessions (Table S2b). In addition, two *PHAS* genes were annotated as *SGS3* and *DCL2* (Table 1). In *Arabidopsis thaliana*, AGO1, SGS3, and DCL2 are key proteins in the phasiRNA biosynthesis pathway [44]. phasiRNAs generated from these loci may therefore affect secondary phasiRNA production.

Compared to the same tissue from C08, the abundances of four phasiRNAs from *NTL9* genes, three phasiRNAs from genes encoding proteins with the *NB-ARC* domain and 1 phasiRNA from the *AFB3* gene were elevated in W05 cotyledons (Table 2). The overexpression of *NAC* factors in *Arabidopsis* and rice was shown to improve tolerance to biotic and abiotic stress [45]. W05 also exhibited strong salt tolerance in the early growth period [46]. Moreover, the *AFB3* protein is an auxin receptor, and in *Arabidopsis*, *AFB3* coordinates primary and lateral root growth and the *NAC4* transcription factor acts downstream of *AFB3* to control root nitrate responses [47]. Seeing as the *AFB3* protein acts as a crucial receptor in the auxin-signaling pathway, which affects root elongation, it is reasonable to observe a higher abundance of this phasiRNA in roots. These possible links gave us hints that phasiRNAs might be related to multiple stress responses, while W05 happened to have strong salt tolerance in the early growth period [46]. Whether these elevated phasiRNAs are related to W05’s responses to salt stress requires further investigation.

The two soybean lines possess different seed sizes and seed coat colors (Figure S5). Seeds of C08 are yellow and are larger than seeds of W05, which are black [21,46]. Large seed size was likely a trait actively selected for over the course of human domestication, and the yellow seed coat color may have been a by-product of this artificial selection. C08 cotyledons are larger in size, but their salt tolerance is much lower than W05 [46]. Larger, heavier seeds contain more nutrients, providing more energy for germination. In phasiRNA profiles, a great number of phasiRNAs were suppressed in W05 germinated roots (Table 2). These phenotypic differences may cause the differences in gene expressions and sRNA profiles between the two accessions during germination.

Interestingly, in both wild and cultivated accessions, three phasiRNA-producing *TIR-NB-LRR* loci (*Glyma.04G219600*, *Glyma.06G285500*, and *Glyma.12G163000*) were preferentially accumulated in leaves and a different *TIR-NB-LRR* phasiRNA-producing locus (*Glyma.06G205100*) was preferentially accumulated in roots, across all three developmental stages (Table 3). Considering roots and leaves face different pathogens in their respective environments, these *TIR-NB-LRR* loci originated, differentially accumulated phasiRNAs may play important roles in immune defense [48,49].

### 3.3. miRNA–phasiRNA Regulation in Soybean

Fifty-two phasiRNAs triggered by 17 miRNAs were identified in accession C08, and 50 phasiRNAs triggered by 15 miRNAs were identified in accession W05 (Table S5a). miR2118, miR390, and miR9761 were unique in C08, and miR1536 was unique in W05. We found the miR2118-induced phasiRNA (Chr03_35271156) was highly accumulated in C08. This secondary small RNA has a high sequence similarity to *TIR-NBS-LRR* mRNAs. In *Medicago truncatula*, some *TIR-NBS-LRR* disease resistance genes were validated as the targets of miR2118 [50]. It is possible that Chr03_35271156 phasiRNAs cleave or inhibit the translation of *TIR-NBS-LRR* mRNAs in order to simultaneously regulate multiple members of the same gene family. For other large plant gene families like PPR, this gene regulation role undertaken by phasiRNA has already been demonstrated [2,51]. In *Arabidopsis* *thaliana*, *TAS3*-derived tasiARFs, which are *trans*-acting small interfering RNAs triggered by miR390, target ARFs and regulate plant growth [52]. miR390 accumulated in both accessions but the corresponding phasiRNAs were only generated in C08. These phasiRNAs may play a role in the development of C08. In general, phasiRNAs can act as amplifiers that downregulate multiple highly homologous target genes.

We applied next-generation sequencing and analyzed 14 small RNA libraries generated from various tissues and developmental stages of wild and cultivated soybean. With these data, we found 458 (443 known and 15 novel) miRNAs to be differentially expressed. Additionally, a statistically rigorous pipeline to identify *PHAS* loci in soybean was developed based on previous work [6], which identified 50 PHAS loci containing 55 phasiRNAs in C08 and 41 PHAS loci containing 46 phasiRNAs in W05 (Figure 4). miRNAs that might trigger the biogenesis of phasiRNAs were also predicted (Table S5). This study provides a global view of sRNAs in C08 and W05. It can be a reference for future functional studies of sRNAs in *Glycine max* and *Glycine soja*.

## 4. Materials and Methods

### 4.1. Plant Materials

The phylogenetic relationship between C08 and W05 was reported previously [21]. C08 is a cultivated soybean from the USA, and is a close relative of Williams 82. W05 is a wild soybean originating from Henan Province, China.

Seed preparation. Since the seed coat of W05 seed is hard, the W05 seeds were scarified by a razor blade before germination to ensure a uniform germination rate. Germinated seedlings, young seedlings, and seedlings samples were collected, and their descriptions are as follows. Germinated seedling. C08 and scarified W05 seeds were germinated in ~70% tap-water-moistened vermiculite in the dark with a lid. After 72 h, the lid was removed and germinated seedlings continued to grow. Four- to five-day-old seedlings were collected as germinated seedlings. Young seedlings. Seven-day-old seedlings from germination were then transferred to a hydroponic system with Hoagland’s solution. Young seedling samples were collected when the primary leaves were fully opened. Seedlings. Seedlings samples were collected when the first set of trifoliate leaves were fully opened. Aerial part, trifoliate and root samples of seedling samples were collected.

### 4.2. RNA Extraction and Sequencing of Small RNA

Total RNA from soybean plants was extracted as previously described [53]. The total RNA was sent to the Beijing Genomics Institute (BGI) (Shenzhen, China). Fourteen small RNA libraries from different developmental stages and tissue types of wild and cultivated soybeans were sequenced using Illumina HiSeq™ 2000 high throughput sequencing technology, as previously described, in order to obtain single-end, directional reads [51].

### 4.3. Reads Pre-Processing and Mapping for Downstream Analyses

As only 2.35% of the 937.5-Mb genome sequences differ between *G. max* and *G. soja* [23], high quality *G. max* genome sequences were used for mapping. After adaptor-trimming, reads were mapped against the soybean genome (Wm82.a2.v1) with different methods for sRNA discovery and expression tabulation purposes. To consider all possible new miRNA/phasiRNA loci, two aligners, Bowtie version 0.12.7 [54] (with -v 0 –k 20 –best –strata –S option) and Soap3-dp [55] were used to identify reads without mismatches and were mapped to either single or multiple positions in the genome, accepting up to 20 valid alignments. For expression tabulation purposes, reads were mapped with Bowtie version 0.12.7 (with -v 0 –k 1 –best option), accepting uniquely mapped reads, as well as one valid alignment per read for multi-mapped reads. The mapping results are summarized in Supplemental Table S1.

### 4.4. Generation of rRNA, tRNA, Repeat Regions, snRNA/snoRNA Annotations

Ribosomal RNA, tRNA, and repeat sequences in the reference genome were annotated by RNAmmer 1.2 [56], tRNAscan-SE 1.23 [57] and RepeatMasker 4.0.5 [58], respectively. Covariance models (CMs) of snRNAs/snoRNAs were extracted from Rfam (12.0) CM [59] using cmfetch of Infernal-1.1 [60], and was used to search against the Wm82.a2.v1 reference genome. Hits with *E*-values below the inclusion threshold of 0.01 were accepted as snRNAs/snoRNAs.

### 4.5. Soybean MicroRNA Discovery and Annotation

The mapped reads for discovery purposes were fed into the miRNA prediction software miR-PREFeR [61]. Predicted miRNA precursors and their corresponding mature miRNAs were annotated by BLASTn [62] against the miRNA precursors in miRBase (release 21) [63], and the best miRBase hit with query coverage ≥90% and identity ≥90% over the length of the matched sequence was considered the same miRNA precursor. Those remaining were considered to be novel miRNA candidates. Predicted miRNAs arising from rRNA, tRNA, repeat regions, snRNA, and snoRNA loci were discarded.

The genomic locations of all known soybean miRNA precursors on soybean genome v1.0 (genome-build accession: NCBI Assembly: GCA_000004515.1) were obtained from miRBase. To determine the location of known miRNAs on the new reference genome assembly Wm82.a2.v1, miRNAs with a 500-bp flanking region on each side were extracted for BLASTn against the reference genome Wm82.a2.v1. Hits with 100% identity and 100% coverage over the flanking regions were accepted as the location of the extracted region in the reference genome, and precursors without a match with these criteria were subjected to the next round of mapping. The process was repeated with 200-bp flanking lengths, 50-bp flanking lengths, and, finally, no flanking regions to determine the genomic location of miRNA genes in the genome assembly Wm82.a2.v1.

### 4.6. Normalization and Differential Gene Expression Calling of miRNAs

To tabulate miRNA expression levels, read counts were tabulated from the expression tabulation purpose mapped reads. The size factor used to normalize the read counts was calculated with the DESeq package [64], and the normalized read counts were then analyzed by the DEGseq package [65] to identify differentially expressed miRNAs. Pairwise library comparisons for each *MIR* gene were performed and *MIR* genes that had a *p*-value <0.001 and an absolute log fold-change ≥2 indicated a differential expression between the two conditions being compared.

### 4.7. Target Prediction of miRNAs

The targets of miRNAs were predicted with psRNATarget [66] using the Wm82.a2.v1 transcripts as a database. The targets predicted with an expectation value ≤3 were considered to be confident predictions.

### 4.8. Prediction of phasiRNA Regions

Reads in the discovery purpose-mapped read set with no more than six mapped positions in the genome were selected for feeding into the phasiRNA prediction algorithm. Tailored for deep sequencing data, a sliding window method was adopted to detect *PHAS* regions, where phasiRNAs were generated based on read mapping patterns [30,67]. In References [30,67], the 231 nt downstream window of each 5’ mapping position was examined and a *p*-values test was performed in the window. The number of windows to be examined equals to the number of read mapping positions on genome, and it was positively correlated with sequencing depth. We assumed that the phasiRNA was likely to be 21 nt long and reduced the window size to be 10 times the length (i.e., 210 nt) of the phasiRNA being tested. Starting from the 5’ position of each read mapped region on genome, we started *p*-values tests [30,67] in the 210 nt window and the window was shifted *s* (*s* = 21) positions at each step towards the 3’ end of the reference genome after each test. Therefore, the number of windows to be examined equals to the total length of read mapped regions divided by 21 and would not increase as sequencing depth deepened. Phase shift (*x*, from 1 to 21) was defined to indicate the position of the phasiRNAs generated from one *PHAS* region relative to the window start position. Thus, different *PHAS* regions predicted from the same window could be distinguished from one another. For each window and each phase shift *x*, a *p*-values test was applied to all mapped reads. If the *p*-value was below 1 × 10^−6^, it implied that on-phase (*x*) *s*-long reads were significantly more abundant than those that were not on-phase (*x*). Thus, a *PHAS* region was called within the window on phase *x*. After all *PHAS* regions were called, we further selected the top 50 highest-ranking candidates, based on phase scores [18].

### 4.9. Quantitative RT-PCR

Quantitative reverse transcription polymerase chain reaction (qRT-PCR) analysis was carried out using cDNA samples on a StepOnePlus Real-Time PCR System (Applied Biosystems, Waltham, CA, USA). cDNAs were synthesized from 4 μg of total RNA by the miRNA First Strand Synthesis Kit (Clontech, CA, USA), and SYBR Green Master Mix (Applied Biosystems) was used in the qPCR experiments. miR1520d was used as the internal control. For every transcript, each cDNA sample was analyzed in triplicate, and the relative transcript abundance was calculated by normalizing to the maximum concentration. The assessment of accumulation comparing different targets was determined by the 2^−ΔΔ*C*t^ method.

## Figures and Tables

**Table 1:** *PHAS* regions and their annotations in soybean.

Chr	PHAS Start	PHAS End	Accession	Gene Name	Homologous Gene	Annotation
Chr02	45,229,200	45,229,892	C08	NA	NA	NA
Chr03	35,270,999	35,271,544	C08	*Glyma.03G136600*	*AT2G41750*	DTW domain-containing protein
Chr04	47,362,862	47,363,575	C08	NA	NA	NA
Chr04	49,035,971	49,037,012	C08,W05	*Glyma.04G219600*	*AT5G36930*	Disease resistance protein (TIR-NB-LRR class) family
Chr05	656,827	657,414	C08	*Glyma.05G006900*	*AT3G14470*	NB-ARC domain-containing disease resistance protein
Chr05	38,268,854	38,269,817	C08,W05	*Glyma.05G198500*	*AT5G23570*	SGS3, XS zinc finger domain-containing protein
Chr06	11,932,996	11,934,011	C08,W05	*Glyma.06G146200*	*AT5G36930*	Disease resistance protein (TIR-NB-LRR class) family
Chr06	19,392,730	19,393,674	C08,W05	*Glyma.06G205100*	*AT5G17680*	Disease resistance protein (TIR-NB-LRR class), putative
Chr06	39,170,442	39,171,407	W05	*Glyma.06G239000*	*AT5G17680*	Disease resistance protein (TIR-NB-LRR class), putative
Chr06	43,287,288	43,287,977	C08,W05	*Glyma.06G254300*	*AT3G14470*	NB-ARC domain-containing disease resistance protein
Chr06	47,410,720	47,411,412	C08,W05	*Glyma.06G285500*	*AT5G17680*	Disease resistance protein (TIR-NB-LRR class), putative
Chr07	4,028,350	4,029,084	C08,W05	*Glyma.07G048000*	*AT4G35580*	NTL9 NAC transcription factor-like 9
Chr08	41,923,481	41,923,921	C08,W05	*Glyma.08G301200*	*AT5G36930*	Disease resistance protein (TIR-NB-LRR class) family
Chr08	41,923,963	41,924,634	C08	*Glyma.08G301200*	*AT5G36930*	Disease resistance protein (TIR-NB-LRR class) family
Chr09	2,041,836	2,042,507	C08,W05	*Glyma.09G025300*	*AT3G03300*	ATDCL2,DCL2 dicer-like 2
Chr09	2,737,112	2,737,899	C08,W05	*Glyma.09G032400*	NA	NA
Chr09	47,565,020	47,565,481	W05	*Glyma.09G256600*	*AT3G22470*	Pentatricopeptide repeat (PPR) superfamily protein
Chr11	30,572,116	30,573,147	C08,W05	*Glyma.11G212800*	*AT3G14460*	LRR and NB-ARC domains-containing disease resistance protein
Chr12	31,231,341	31,232,533	C08,W05	*Glyma.12G163000*	*AT1G31540*	Disease resistance protein (TIR-NB-LRR class) family
Chr12	39,530,806	39,531,225	C08	*Glyma.12G236500*	*AT4G27220*	NB-ARC domain-containing disease resistance protein
Chr13	19,268,532	19,269,329	C08	NA	NA	NA
Chr13	29,861,566	29,862,427	C08	*Glyma.13G184800*	*AT3G14460*	LRR and NB-ARC domains-containing disease resistance protein
Chr13	29,875,305	29,876,627	C08,W05	*Glyma.13G184900*	*AT3G14470*	NB-ARC domain-containing disease resistance protein
Chr13	30,174,985	30,175,782	C08,W05	*Glyma.13G187900*	*AT3G14470*	NB-ARC domain-containing disease resistance protein
Chr13	30,207,736	30,208,554	C08,W05	*Glyma.13G188300*	*AT3G14470*	NB-ARC domain-containing disease resistance protein
Chr13	30,389,026	30,390,362	C08,W05	*Glyma.13G190300*	*AT3G14470*	NB-ARC domain-containing disease resistance protein
Chr13	30,664,791	30,665,882	C08	*Glyma.13G193300*	*AT3G14470*	NB-ARC domain-containing disease resistance protein
Chr13	30,727,236	30,728,889	C08,W05	*Glyma.13G194100*	*AT3G14470*	NB-ARC domain-containing disease resistance protein
Chr13	30,915,467	30,916,705	C08,W05	*Glyma.13G195600*	*AT3G14460*	LRR and NB-ARC domains-containing disease resistance protein
Chr14	3,878,256	3,878,843	C08	NA	NA	NA
Chr15	43,699,964	43,700,551	C08,W05	*Glyma.15G232400*	*AT5G41540*	Disease resistance protein (TIR-NB-LRR class) family
Chr15	43,746,080	43,746,499	C08	*Glyma.15G232800*	*AT3G14460*	LRR and NB-ARC domains-containing disease resistance protein
Chr15	44,546,466	44,547,735	C08,W05	NA	NA	NA
Chr16	1,452,507	1,453,157	C08,W05	*Glyma.16G016600*	*AT4G35580*	NTL9 NAC transcription factor-like 9
Chr16	1,453,270	1,453,905	C08	*Glyma.16G016600*	*AT4G35580*	NTL9 NAC transcription factor-like 9
Chr16	1,461,742	1,462,416	C08,W05	*Glyma.16G016700*	*AT4G35580*	NTL9 NAC transcription factor-like 9
Chr16	1,462,506	1,463,177	C08	*Glyma.16G016700*	*AT4G35580*	NTL9 NAC transcription factor-like 9
Chr16	2,941,895	2,942,482	C08,W05	*Glyma.16G031100*	*AT2G46100*	Nuclear transport factor 2 (NTF2) family protein
Chr16	4,855,988	4,856,743	C08,W05	*Glyma.16G050500*	*AT1G12820*	AFB3 auxin signaling F-box 3 protein
Chr16	5,770,754	5,771,618	C08,W05	*Glyma.16G058900*	NA	NA
Chr16	10,648,349	10,648,999	C08,W05	*Glyma.16G087100*	*AT5G36930*	Disease resistance protein (TIR-NB-LRR class) family
Chr16	30,784,351	30,785,128	C08,W05	*Glyma.16G147100*	NA	NA
Chr16	32,109,362	32,110,117	C08,W05	*Glyma.16G161900*	*AT1G12775*	Pentatricopeptide repeat (PPR) superfamily protein
Chr16	32,167,989	32,168,613	C08	*Glyma.16G162800*	*AT1G62670*	RPF2 RNA processing factor 2
Chr16	32,182,422	32,182,883	C08	*Glyma.16G163100*	*AT1G12700*	ATP binding;nucleic acid binding;helicases
Chr16	32,453,347	32,453,892	W05	*Glyma.16G165400*	*AT1G12300*	Tetratricopeptide repeat (TPR)-like superfamily protein
Chr16	33,378,642	33,379,535	W05	*Glyma.16G173200*	NA	NA
Chr16	35,681,818	35,682,321	W05	*Glyma.16G194800*	*AT1G63130*	Tetratricopeptide repeat (TPR)-like superfamily protein
Chr16	35,687,005	35,687,634	C08,W05	*Glyma.16G195000*	*AT1G12775*	Pentatricopeptide repeat (PPR) superfamily protein
Chr16	35,692,915	35,693,523	C08,W05	*Glyma.16G195100*	*AT1G12700*	ATP binding;nucleic acid binding;helicases
Chr16	35,883,305	35,883,850	W05	*Glyma.16G197600*	*AT1G62910*	Pentatricopeptide repeat (PPR) superfamily protein
Chr16	36,088,255	36,088,695	C08,W05	*Glyma.16G199700*	*AT1G62930*	Tetratricopeptide repeat (TPR)-like superfamily protein
Chr16	36,970,009	36,970,575	C08	*Glyma.16G210600*	*AT5G36930*	Disease resistance protein (TIR-NB-LRR class) family
Chr17	40,464,414	40,465,001	C08,W05	*Glyma.17G249500*	NA	NA
Chr19	34,719,617	34,720,288	C08,W05	*Glyma.19G100200*	*AT1G12820*	AFB3 auxin signaling F-box 3 protein
Chr19	40,040,875	40,041,548	C08,W05	NA	NA	NA
Chr20	31,854,969	31,855,514	W05	NA	NA	NA

NA: Not found.

**Table 2:** Changes in the abundances of phasiRNAs in W05 (wild) and C08 (cultivated) soybeans.

Gene Name	Annotation	Number of phasiRNAs	Fold Change of Expression Level (W05/C08)						
			Ga	Gr	Ya	Yr	Sp	Sr	St
*Glyma.04G219600*	Disease resistance protein (TIR-NB-LRR class)	4	1.1	0.4	0.6	1.1	0.7	1.2	0.5
*Glyma.06G146200*	Disease resistance protein (TIR-NB-LRR class)	4	1.4	0.4	0.9	1.1	0.3	0.6	0.3
*Glyma.06G205100*	Disease resistance protein (TIR-NB-LRR class)	2	1.1	0.3	1.4	0.8	1.0	1.5	0.4
*Glyma.06G285500*	Disease resistance protein (TIR-NB-LRR class)	1	1.8	0.5	1.6	1.5	1.5	1.2	1.1
*Glyma.08G301200*	Disease resistance protein (TIR-NB-LRR class)	1	0.9	0.5	0.5	0.7	0.6	1.0	0.4
*Glyma.12G163000*	Disease resistance protein (TIR-NB-LRR class)	2	0.6	0.5	0.9	1.1	1.3	1.1	0.6
*Glyma.13G187900*	NB-ARC domain-containing disease resistance protein	4	3.6	2.2	2.5	2.2	1.5	2.3	3.5
*Glyma.11G212800*	LRR and NB-ARC domains-containing disease resistance protein	1	0.6	0.5	0.6	1.0	0.8	1.5	0.5
*Glyma.07G048000*	NTL9 NAC transcription factor-like 9	4	2.0	1.9	1.8	2.5	1.6	3.2	1.7
*Glyma.16G016600*	NTL9 NAC transcription factor-like 9	1	1.1	0.3	0.4	1.0	0.3	1.1	0.3
*Glyma.09G025300*	ATDCL2, DCL2	2	1.0	0.3	0.9	1.2	0.6	1.4	1.0
*Glyma.16G050500*	AFB3 auxin signaling F-box 3	1	2.7	0.9	1.0	0.8	0.5	0.7	1.3
*Glyma.16G161900*	Pentatricopeptide repeat (PPR) superfamily protein	1	1.0	0.5	0.8	1.0	0.6	1.2	0.7
*Glyma.16G058900*	NA	3	1.0	0.4	0.9	1.0	0.6	1.1	0.5
*Glyma.16G147100*	NA	3	0.9	1.4	0.6	1.0	0.4	1.1	0.6
*Chr15_44546856_44547257*	NA	4	0.6	0.2	0.6	0.8	0.4	0.9	0.6
*Glyma.09G032400*	NA	5	1.1	0.5	0.7	0.8	0.7	1.0	0.5

Ga: Germinated seedling aerial parts. Gr: Germinated seedling roots. Ya: Young seedling aerial parts. Yr: Young seedling roots. Sp: Seedling primary leaves. Sr: Seedling roots. St: Seedling trifoliates. Red: Fold change ≤0.5 (higher abundance in C08). Green: Fold change ≥2 (higher abundance in W05).

**Table 3:** Differential amounts of phasiRNAs in the *PHAS* regions in leaves versus roots.

Gene Name	Annotation	Number of phasiRNA	Leaf/Root (Germinated Seedlings)		Leaf/Root (Young Seedlings)		Leaf/Root (Seedlings)		Leaf/Root (Three Stages)	
			C08	W05	C08	W05	C08	W05	C08	W05
*Glyma.04G219600*	Disease resistance protein (TIR-NB-LRR class)	4	2.2	5.4	2.6	1.6	5.3	3.9	2.9	3.0
*Glyma.06G146200*	Disease resistance protein (TIR-NB-LRR class)	4	0.8	1.8	1.2	1.0	2.9	1.2	1.4	1.3
*Glyma.06G205100*	Disease resistance protein (TIR-NB-LRR class)	2	0.04	0.13	0.01	0.01	0.02	0.02	0.03	0.04
*Glyma.06G285500*	Disease resistance protein (TIR-NB-LRR class)	1	7.1	27.9	6.1	6.8	10.6	13.1	7.3	12.0
*Glyma.08G301200*	Disease resistance protein (TIR-NB-LRR class)	1	0.1	0.3	0.6	0.4	1.5	1.1	0.5	0.5
*Glyma.12G163000*	Disease resistance protein (TIR-NB-LRR class)	2	19.9	34.2	7.0	6.9	12.6	15.6	11.1	11.2
*Glyma.13G187900*	NB-ARC domain-containing disease resistance protein	4	1.1	1.4	1.4	1.6	4.8	2.7	2.2	1.9
*Glyma.11G212800*	LRR and NB-ARC domains-containing disease resistance protein	1	1.2	1.3	0.7	0.4	1.7	1.0	1.2	0.8
*Glyma.07G048000*	NTL9 NAC transcription factor-like 9	4	1.3	1.2	0.4	0.3	1.0	0.6	0.8	0.6
*Glyma.16G016600*	NTL9 NAC transcription factor-like 9	1	1.0	3.2	1.2	0.6	2.5	0.7	1.5	1.2
*Glyma.09G025300*	ATDCL2, DCL2	2	0.4	1.4	0.6	0.5	1.4	0.6	0.7	0.7
*Glyma.16G050500*	AFB3 auxin signaling F-box 3	1	0.1	0.3	0.3	0.4	0.7	0.5	0.5	0.4
*Glyma.16G161900*	Pentatricopeptide repeat (PPR) superfamily protein	1	3.4	7.5	2.7	2.3	6.8	3.1	3.9	3.9
*Glyma.16G058900*	NA	3	0.8	1.6	1.1	0.9	2.3	1.4	1.1	1.3
*Glyma.16G147100*	NA	3	0.4	0.9	1.0	0.5	1.6	0.8	0.8	0.7
*Chr15_44546856_44547257*	NA	4	1.3	3.8	3.0	2.2	5.8	2.6	2.4	2.9
*Glyma.09G032400*	NA	5	1.3	2.0	3.1	2.7	4.4	2.8	2.5	2.5

Red: Fold change ≤0.5 (higher abundance in the roots); Green: Fold change ≥2 (high abundance in the leaves).

## Supplementary Materials

Supplementary materials can be found at www.mdpi.com/1422-0067/17/12/2043/s1.
