# Supplementary material for: Comparison of Small RNA Profiles of Glycine max and Glycine soja at Early Developmental Stages
Source: Int J Mol Sci. 2016 Dec 6;17(12):2043. doi: 10.3390/ijms17122043 (PMC5187843; doi:10.3390/ijms17122043)
Supplement: Supplementary file 1 [file ijms-17-02043-s001.zip › ijms-160957-Supplementary Materials/ijms-160957-SI.pdf]

Supplementary Materials: Comparison of Small RNA Profiles of *Glycine max* and *Glycine soja* at Early Developmental Stages

Yuzhe Sun, Zeta Mui, Xuan Liu, Aldrin Kay-Yuen Yim, Hao Qin, Fuk-Ling Wong, Ting-Fung Chan, Siu-Ming Yiu, Hon-Ming Lam and Boon Leong Lim

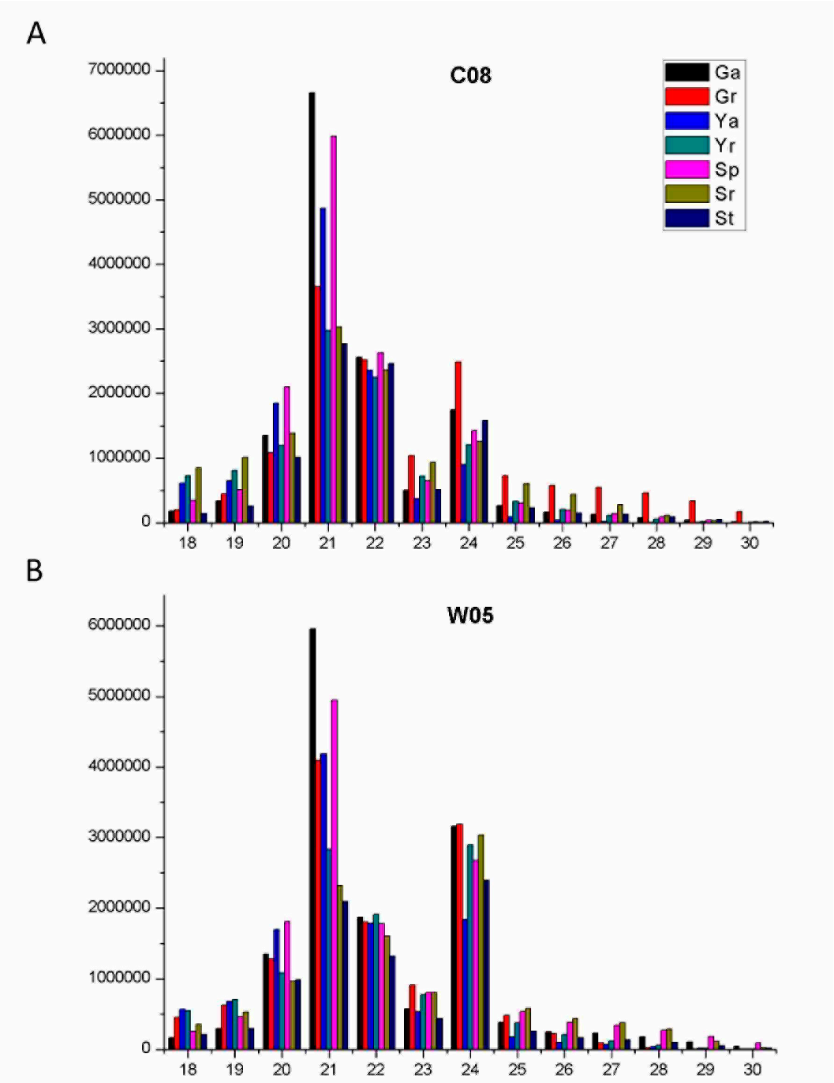

Figure S1. Length distributions of sRNA. The sRNAs of 7 samples each of a cultivated soybean accession (C08) and a wild accession (W05) were sequenced. *X*-axes: Length of reads (nt). *Y*-axes: Total number of reads. (**A**) Length distributions of C08 sRNAs; (**B**) Length distributions of W05 sRNAs. Ga: Germinated seedling cotyledons; Gr: Germinated seedling roots; Ya: Young seedling leaves; Yr: Young seedling roots; Sp: Seedling primary leaves; Sr: Seedling roots; St: Seedling trifoliates.

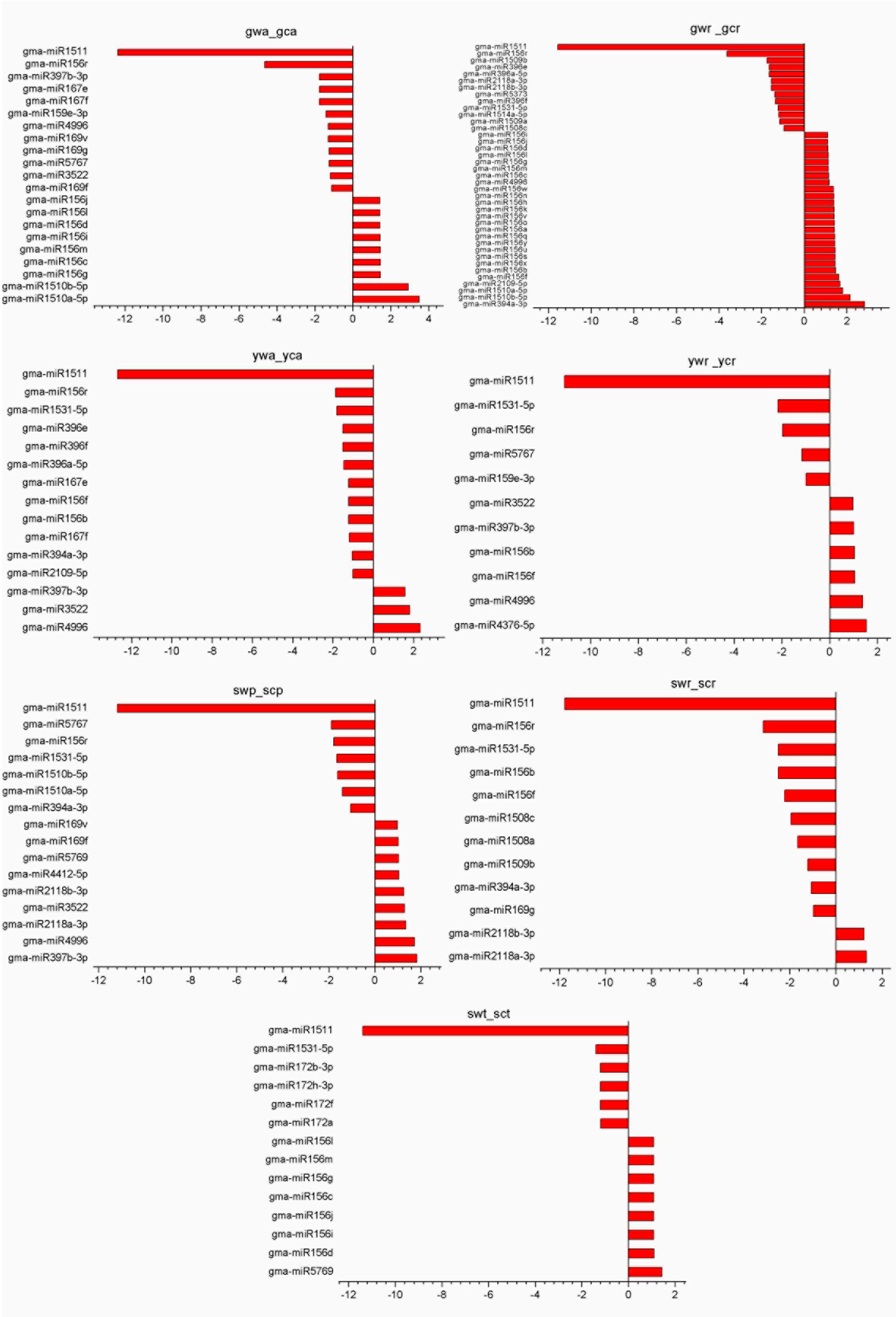

Figure S2. Pairwise comparisons of miRNA expressions between corresponding samples of C08 and W05. The same tissues from different accessions were compared (W05 samples/C08 samples). miRNAs of which fold-change $-1 < Log_2 < 1$ were removed from the dataset.

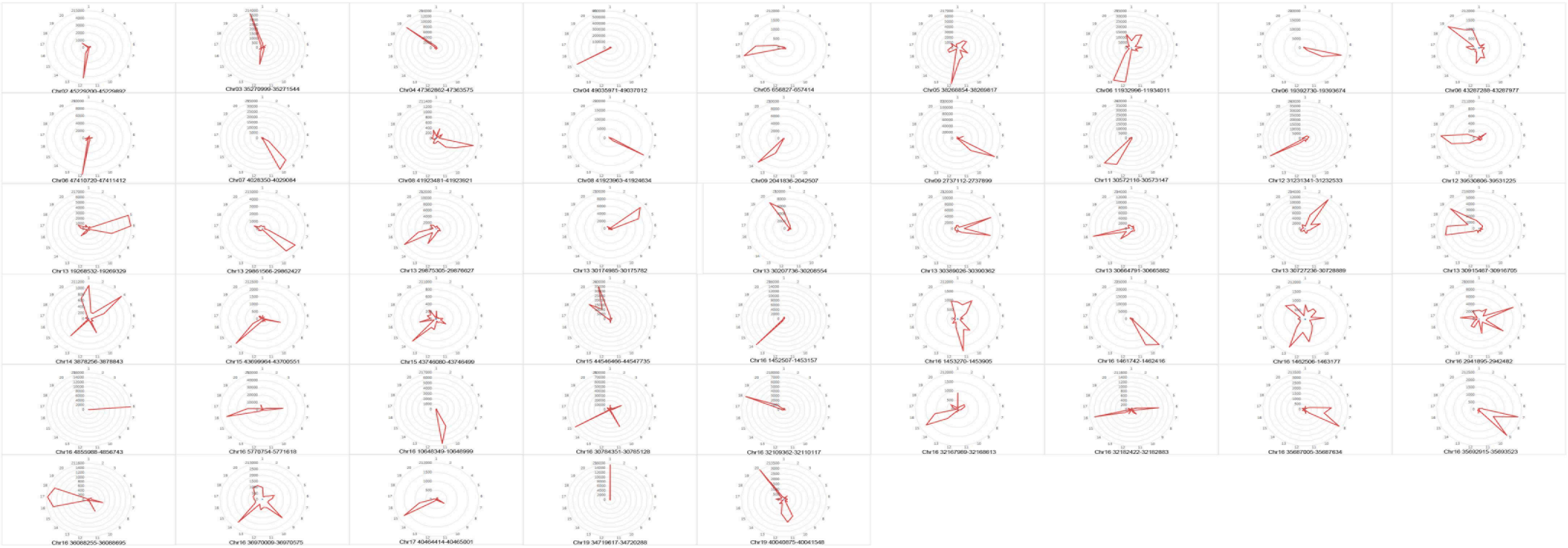

Figure S3. Phase check of *PHAS* regions of C08. Twenty-one-nucleotide sRNA reads in each *PHAS* region of C08 were plotted as radar maps. The needles pointed to the start positions of sRNAs. If a needle pointed to more than two directions, the corresponding sRNA in the region was discarded.

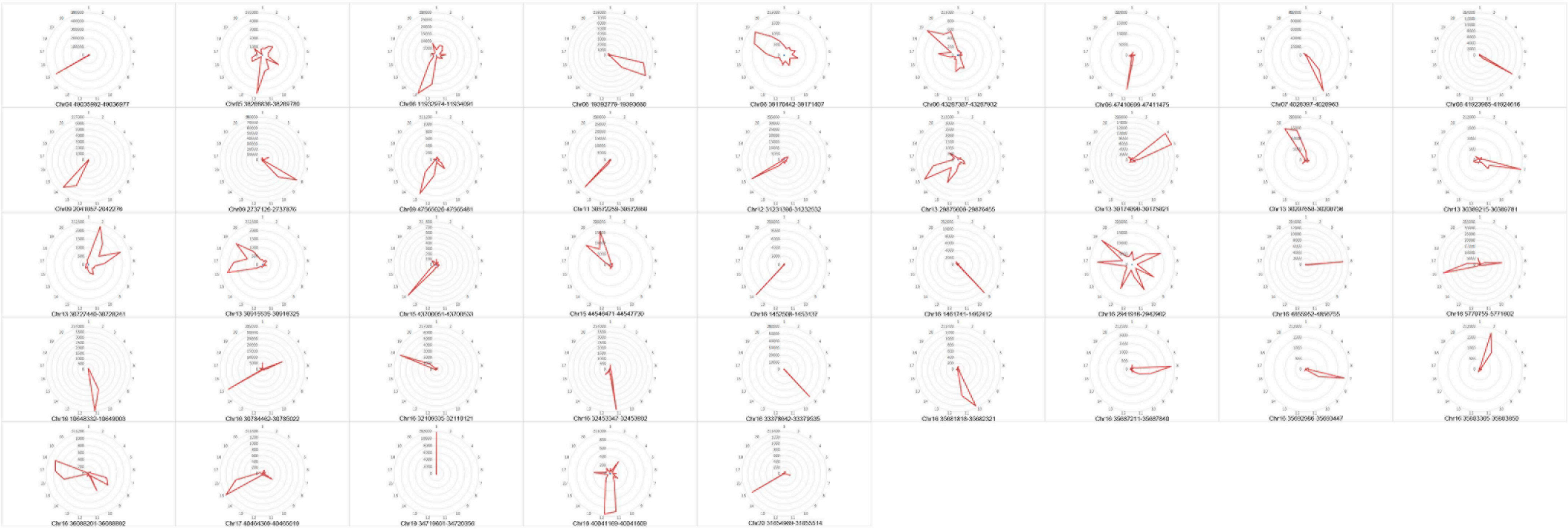

Figure S4. Phase check of *PHAS* regions of W05. Twenty-one-nucleotide sRNA reads in each *PHAS* region of C08 were plotted as radar maps. The needles pointed to the start positions of sRNAs. If a needle pointed to more than two directions, the corresponding sRNA in the region was discarded.

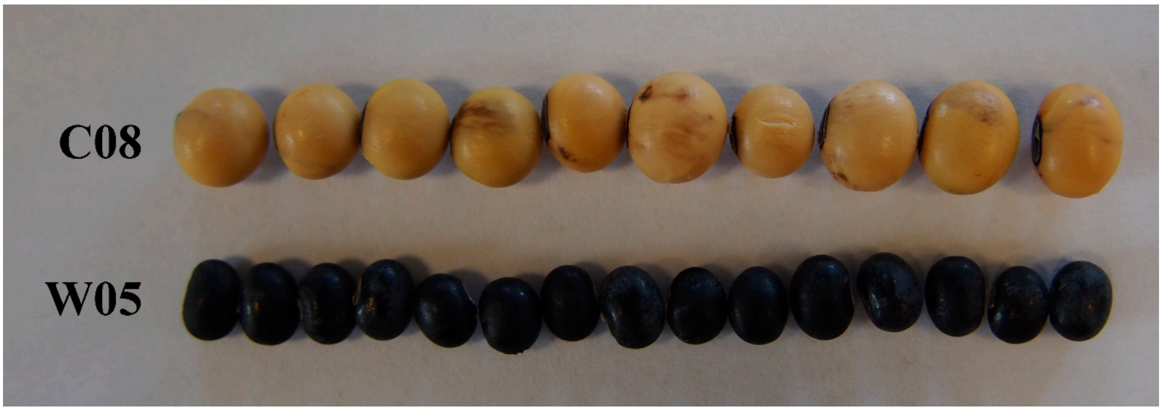

Figure S5. Seeds of wild and cultivated soybeans. Black seeds of wild soybeans (W05) are smaller than yellow seeds of cultivated soybeans (C08).
